# Gender role stereotypes, patriarchal attitudes, and cognitive function in the elderly rural Korean population: a cross-sectional study

**DOI:** 10.4178/epih.e2021023

**Published:** 2021-04-07

**Authors:** Hye Rin Choi, Byeonggwan Ha, Ye Jin Jeon, Yoosik Youm, Hyeon Chang Kim, Sun Jae Jung

**Affiliations:** 1 Healthcare Data Promotion Division, Bureau of Health Industry, Ministry of Health and Welfare, Sejong, Korea; 2 Yonsei University College of Medicine, Seoul, Korea; 3 Department of Public Health, Yonsei University Graduate School, Seoul, Korea; 4 Department of Sociology, Yonsei University College of Sociology, Seoul, Korea; 5 Department of Preventive Medicine, Yonsei University College of Medicine, Seoul, Korea

**Keywords:** Gender role, Stereotyping, Cognitive dysfunction, Geriatrics, Public health

## Abstract

**OBJECTIVES:**

We analyzed whether gender role stereotypes (GRS) and patriarchal attitudes are associated with cognitive function in an elderly community.

**METHODS:**

We analyzed data from 580 people enrolled in the Korean Social Life, Health, and Aging Project. The degrees to which respondents held stereotypical beliefs about gender roles and had patriarchal mindsets were measured using a questionnaire. Based on participants’ responses, we divided respondents into 2 groups—those with conservative mindsets and those with open mindsets—according to the median score for each of the 2 variables. Cognitive function was assessed using the Mini-Mental State Examination, Korean version (MMSE-K). Cognitive impairment was defined as an MMSE-K score ≤21 points. Multivariable logistic regression was performed, adjusting for gender, age, socio-demographic and lifestyle factors, and social network size. Age and lifestyle factors were stratified.

**RESULTS:**

Compared to those with open mindsets, those with conservative mindsets regarding gender roles and patriarchal norms had adjusted odds ratios of 1.88 (95% confidence interval [CI], 1.11 to 3.19) and 1.67 (95% CI, 1.00 to 2.79) for cognitive impairment, respectively. In the stratified analysis, subgroups with younger age and a good lifestyle maintained a protective association with cognitive impairment.

**CONCLUSIONS:**

GRS and a patriarchal mindset were marginally significantly associated with cognitive impairment among women later in life.

## INTRODUCTION

More than 50 million people worldwide have dementia, and that number is estimated to triple by 2050 [1,2]. The rapid growth of the elderly population and changes in familial structure are increasing the burden of dementia [3]. Previous studies investigating gender differences in cognitive function among the elderly attributed these differences to biological factors such as estrogen or apolipoprotein E [4]. However, more recent studies have focused on psychosocial aspects instead, such as education and participation in the labor force [5].

Gender role stereotypes (GRS) refer to the general social expectations placed on members of particular genders. Their cognitive and motivational function affects how people perceive and engage in the world they live in [6]. The term “patriarchy” is defined in various ways based on its use in diverse academic fields such as sociology, anthropology, and women’s studies [7-9]. In our study, we defined “patriarchy” as a social and familial structure in which men hold more power than women, which is reflected in wider society [10]. It has been suggested that patriarchal mindsets have a major influence on individual behavior, from everyday family affairs to major life-changing decisions. Patriarchal status is inherited through the men lineage. Patriarchal norms and GRS affect peoples’ lifestyles, and patriarchal beliefs, such as the idea that men should dominate over women, which can influence relationships between people of different genders [9,11]. However, patriarchal norms also include concepts regarding inter-generational relationships, such as the dominance of older generations over younger generations, an emphasis on obedience for children, and familial preference for the eldest son [11]. Previous studies have suggested that attitudes toward traditional gender roles or patriarchal norms can constrain or encourage individuals to make certain choices because of their gender, which thereby influence cognitive function later in life [5,12-14]. Unlike GRS, few studies have investigated the impact of patriarchal attitudes on cognitive function, even though such attitudes are an important factor influencing how people act within the circumstances of their lives [15,16]. Furthermore, most previous studies were conducted in Western countries, and the association between GRS and cognitive impairment has not been fully investigated in an East Asian context. East Asian culture has relatively pronounced patriarchal qualities and Confucian values, which are associated with a higher prevalence of mental disorders [17]. Korea, as an East Asian country, has historically shown a strong cultural tendency toward stereotypical gender roles and patriarchal norms, with those tendencies being most pronounced among the elderly rural population [18]. Therefore, we aimed to investigate the association of GRS and patriarchal mindsets with cognitive impairment in a rural Korean population.

## MATERIALS AND METHODS

### Study population

**Figure 1.**
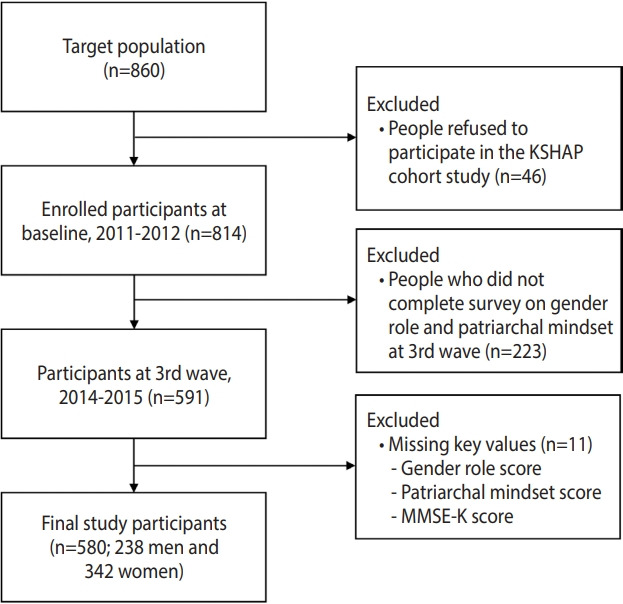
Flow chart of the study population. KSHAP, Korean Social Life, Health, and Aging Project; MMSE-K, Mini-Mental State Examination, Korean version.

The data for this study were collected from the Korean Social Life, Health, and Aging Project (KSHAP) cohort study initiated in 2011. The KSHAP study recruited individuals aged 60 or older, and their spouses, who lived in Township K, Ganghwa Island, Incheon, Korea [19]. With the aid of township officers, after performing a pilot study, a total of 860 people were identified as the target population of KSHAP [20]. To obtain consent and administer questionnaires, we visited participants’ homes individually. In total, 814 of the 860 community-dwelling adults (response rate, 94.7%) participated in the study and completed questionnaire surveys from December 2011 to July 2012 [21]. After the baseline study began, we conducted additional follow-up surveys and health examinations 4 times until 2019. In 2017, we conducted an additional baseline-only study on 948 participants living in Township L, Ganghwa Island, Incheon, Korea. We collected data on GRS in the same way as in Township K. Since the questionnaires about GRS and patriarchal attitudes were only included in the third wave in Township K, we conducted a cross-sectional analysis using only the thirdwave data (n= 591). We also used data on GRS in Township L to replicate the main results in Township K. We excluded 11 participants from the study due to missing key variables such as total scores for views on gender roles (n= 8), patriarchal attitudes (n= 1), and cognitive function (n= 2), leaving 580 people (238 men and 342 women) for the final analysis (Figure 1).

### Measurements

Our trained personnel interviewed participants using standardized questionnaire surveys according to a pre-defined protocol. We used the questionnaires to obtain information on socio-demographic characteristics including age, education, economic and marital status, health behaviors, medical history, depressive symptoms, social network size, quality of the spousal relationship, and network constraints of spouse. We measured the quality of the spousal relationship by calculating the frequency of communication and degree of emotional intimacy between spouses. Spouse constraints refer to the high-density networks surrounding a married couple [22]. We calculated network constraints of spouse based on social network information using a network approach, as per previous studies [23,24]. Cognitive function was assessed using the Mini-Mental State Examination, Korean version (MMSE-K), which has been widely applied in epidemiological studies [25]. The scores of the MMSE-K range from 0 points to 30 points. Cognitive impairment was defined as an MMSE-K score of ≤ 21 points, based on the cut-off recommended in previous studies [26].

We asked about participants’ beliefs regarding gender roles using a questionnaire developed by combining 2 parts of the International Social Survey Programme (ISSP) 2002 Family and Changing Gender Roles III module. The ISSP is an ongoing cross-national collaboration to develop surveys for important social science research [27]. Since this survey has already been used in numerous studies, it has high validation levels, and 1 of its main topics is GRS in the family [28]. Therefore, we used 2 statements from the ISSP questionnaire to measure beliefs regarding stereotypical gender roles in our study population. The statements on GRS were as follows: (1) “Both the man and woman should contribute to household income,” and (2) “A man’s job is to earn money, a woman’s job is to look after the home and family.” Each statement had five possible responses: “strongly agree,” “agree,” “neither agree nor disagree,” “disagree,” and “strongly disagree.” Responses were scored using a corresponding numerical scale ranging from 1 (“strongly agree”) to 5 (“strongly disagree”) points. Possible total scores ranged from 2 points to 10 points. People with lower scores had a more conservative mindset regarding GRS, while those with higher scores had a more open mindset. Thus, for statistical analysis, we divided participants into 2 groups—conservative mindset and open mindset—according to the median score total to analyze beliefs about GRS.

Using a similar method, we conducted another questionnaire to measure patriarchal attitudes using parts from the East Asian Social Survey (EASS) 2006 family module. The EASS is a social survey project and one of the few internationally-coordinated social survey data collection efforts focused on East Asia [29]. The survey examined the traditional patriarchal attitudes of Korean, Chinese, Japanese, and Taiwanese respondents. We selected the following 5 statements from the survey to measure patriarchal attitudes: (1) “the authority of the father in a family should be respected under any circumstances,” (2) “children must make efforts to do something that would bring honor to their parents,” (3) “the eldest son should inherit a larger share of the property,” (4) “a child who has taken good care of parents should inherit a larger share of the property,” and (5) “to continue the family line, one must have at least one son.” Participants selected 1 of 7 possible responses: “strongly agree,” “fairly agree,” “somewhat agree,” “neither agree nor disagree,” “somewhat disagree,” “fairly disagree,” and “strongly disagree.” Responses were measured using a corresponding numerical scale ranging from 1 (“strongly agree”) to 7 (“strongly disagree”) points. Possible total scores for patriarchal mindset ranged from 5 points to 35 points. As for GRS scores, a lower point total indicated a more conservative patriarchal mindset, and a higher total indicated a more open mindset. For statistical analysis, participants were divided into open mindset and conservative mindset groups according to the median score total for patriarchal attitudes. Using binary variables allowed us to concentrate more on the implications of the results. For the model used to assess the association between GRS, patriarchal attitudes, and cognitive function, we selected covariates based on previous studies, including age [30], education [31], household income [32], hypertension [33], physical activity [34], depressive symptoms [35], social network size [36], smoking status [37], and drinking status [38].

### Statistical analysis

The differences between socio-demographic characteristics according to beliefs regarding GRS and patriarchal attitudes were analyzed using the independent t-test for continuous variables and the chi-square test for categorical variables. Continuous variables that followed a normal distribution were shown as mean and standard deviation, whereas skewed variables were expressed as median and interquartile range. Categorical variables were described as numbers and percentages. We divided views on GRS and patriarchal attitudes into 2 groups—conservative mindset and open mindset—according to the median scores for each. To evaluate the independent associations of views on GRS and patriarchal attitudes with cognitive impairment, we performed multiple logistic regression analyses of men and women, employing unadjusted and fully adjusted models. The full model was adjusted for gender, age, household income, education, hypertension, physical activity, depressive symptoms, social network size, smoking status, and drinking status. Furthermore, we performed subgroup analyses of groups divided by quality of spousal relationship, network constraints of spouse [39], age [30], and lifestyle [37,38], based on the analyses of previous papers. Because GRS and patriarchal attitudes are usually influenced by social relationships and environment, the impacts of age, lifestyle, and social network needed to be investigated to determine whether they interacted with or were effect modifiers for associations between views on GRS, patriarchal attitudes, and cognitive impairment. The quality of spousal relationship and network constraints of spouse were only divided according to their median values for analyzing GRS, and not for patriarchal attitudes, since GRS was based on the spousal relationship. We divided age into 2 groups according to the median age of 73. We divided frequency of physical activity into 3 groups: less than once per week, 1-4 times per week, and 5 or more times per week. Social network size was classified into 2 groups according to the median level of 3. Depressive symptoms were assessed using the Center for Epidemiologic Studies Depression scale. We used the variables for drinking and smoking status to delineate lifestyle behaviors into 2 groups, namely “good” and “bad.” We operationally defined the “good lifestyle” group as consisting of those who reported they were never, or were former but not current, drinkers and smokers. Current drinkers or smokers comprised the “bad lifestyle” group. All analyses were performed using SAS version 9.4 (SAS Institute Inc., Cary, NC, USA). Statistical significance was defined as a 2-sided p-value of less than 0.05.

### Ethics statement

The Institutional Review Board of Yonsei University (YUIRB-2011-012-01) approved the protocol of the KSHAP study. All methods were performed in accordance with relevant guidelines and regulations. Informed consent was obtained from all participants.

## RESULTS

Table 1 presents the general characteristics of the study population according to beliefs regarding GRS and patriarchal attitudes. The mean ages were significantly higher for groups with conservative mindsets regarding GRS or patriarchal norms than for open-minded groups. The proportion of respondents with an education level lower than elementary school was significantly higher for conservative groups than for open-minded groups. The mean MMSE-K scores were significantly lower for conservative groups compared to the open-minded groups. We also provide data on differences in general characteristics by gender in Supplementary Material 1 and differences in general characteristics by cognitive status in Supplementary Material 2.

**Figure 2.**
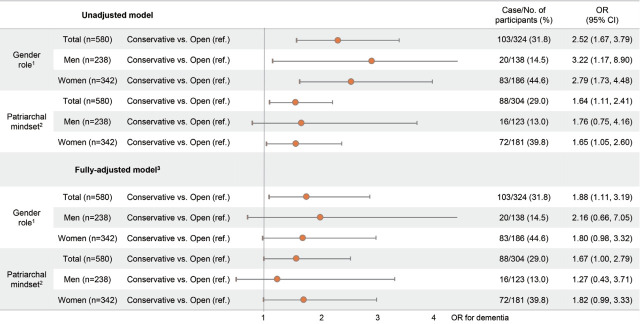
Odds ratios (ORs) for cognitive impairment among the total study population, men only, and women only in unadjusted and fullyadjusted models. CI, confidence interval; ref., reference. ^1^ People with open attitude to gender role stereotype were defined as those with over the median score, 6 points, of gender role questionnaires. ^2^ People with open attitude to patriarchal mindset were defined as those with over the median score, 13 points, of patriarchy questionnaires. ^3^ Adjusted for gender, age, household income, education, hypertension, physical activity, depressive symptoms, social network size, smoking and drinking status.

Figure 2 shows the association of beliefs regarding GRS and patriarchal attitudes with cognitive impairment in total, in men only, and in women only. Of the total number of participants, the unadjusted odds ratio (OR) of a conservative mindset toward gender roles for having cognitive impairment was 2.52 (95% confidence interval [CI], 1.67 to 3.79) compared to an open mindset. After adjusting for gender, age, household income, education, hypertension, physical activity, depressive symptoms, social network size, smoking status, and drinking status, the adjusted OR for cognitive impairment was 1.88 (95% CI, 1.11 to 3.19) in the group with a conservative mindset toward GRS compared to the open-minded group. Furthermore, the adjusted ORs for cognitive impairment were 2.16 (95% CI, 0.66 to 7.05) and 1.80 (95% CI, 0.98 to 3.32) for men and women, respectively, in the group with a conservative mindset toward GRS. Moreover, there was no significant interaction between beliefs regarding GRS and gender (p= 0.696). Regarding patriarchal attitudes, among all participants, the unadjusted OR for cognitive impairment was 1.64 (95% CI, 1.11 to 2.41) for people with a conservative mindset toward patriarchy compared to the group with an open mindset. After adjusting for potential confounders, the ORs for cognitive impairment were 1.27 (95% CI, 0.43 to 3.71) and 1.82 (95% CI, 0.99 to 3.33) in men and women, respectively, in the group with a conservative mindset toward patriarchy. The interaction between patriarchal attitudes and gender was not significant (p= 0.529). To replicate these results, we performed another robust test using an independent sample consisting of participants living in Township L. As shown in Supplementary Material 3, we found that the significant association between beliefs regarding GRS and cognitive impairment was consistent with our main results for participants living in Township K. To support the main results in Figure 2, we also examined the associations of beliefs regarding GRS and patriarchal attitudes with continuous MMSE-K scores using linear regression analysis, shown in Supplementary Materials 3 and 4, respectively. People with conservative beliefs regarding GRS were more likely to have low MMSE-K scores than those in the open-minded group. Similarly, those with a conservative mindset toward patriarchal norms were also more likely to have low MMSE-K scores, but the results were not statistically significant.

**Figure 3.**
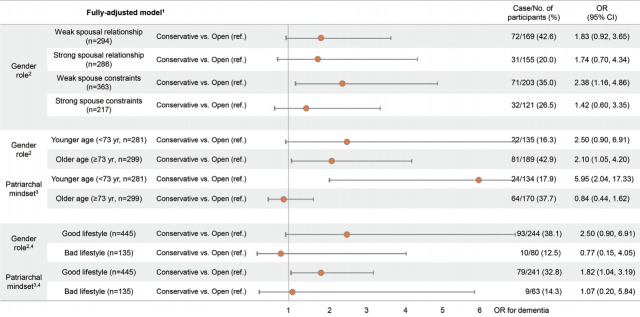
Odds ratios (ORs) for cognitive impairment among groups stratified by quality of spousal relationship, spouse constraints, age, and lifestyle in the fully-adjusted model. Cl, confidence interval; ref., reference. ^1^ Adjusted for gender, age, household income, education, hypertension, physical activity, depressive symptoms, social network size, smoking and drinking status. ^2^ People with open mind to gender role were defined as those with over the median score, 6 points, of gender role questionnaires. ^3^ People with open mind to patriarchal mindset were defined as those with over the median score, 13 points, of patriarchy questionnaires. ^4^ Good lifestyle group consists of never or former drinkers and smokers; bad lifestyle group consists of current drinkers or current smokers.

Figure 3 shows the association of beliefs regarding GRS and patriarchal attitudes with cognitive impairment from the subgroup analysis of age and lifestyle behaviors. In addition, we analyzed the associations between beliefs regarding GRS and cognitive impairment in subgroups defined according to the quality of spousal relationship and network constraints of spouse, since the quality of spousal relationship was only related to GRS, and not patriarchal attitudes. For the subgroups with weak spousal relationships and spousal constraints, the fully adjusted ORs for cognitive impairment were 1.83 (95% CI, 0.92 to 3.65) and 2.38 (95% CI, 1.16 to 4.86), respectively, for those with conservative mindsets regarding GRS compared to those with open mindsets. The older subgroup (≥ 73 years old) with a conservative mindset toward GRS had a significantly higher (OR, 2.10; 95% CI, 1.05 to 4.20) for cognitive impairment than older people with open mindsets did. For those with a good lifestyle, the adjusted ORs for cognitive impairment were 2.50 (95% CI, 0.90 to 6.91) and 1.82 (95% CI, 1.04 to 3.19) for groups with conservative mindsets toward GRS and patriarchal norms, respectively, compared to groups with open mindsets. The results for the association between patriarchal attitudes and cognitive impairment in the younger group showed that the adjusted OR for cognitive impairment was significantly higher for those with conservative mindsets toward patriarchal norms. In addition, there was a significant interaction between patriarchal attitudes and age (p= 0.006).

## DISCUSSION

We observed marginally significant associations of beliefs regarding GRS and patriarchal attitudes with cognitive impairment in women after adjusting for potential confounders. Additionally, we found a significant positive association between beliefs regarding GRS and cognitive impairment among groups with weak network constraints of spouse and a high age. After stratifying by age, people with conservative mindsets toward patriarchal norms who were relatively younger had a significantly higher tendency for having cognitive impairment. Thus, we suggest that age is an effect modifier for the association between patriarchal attitudes and cognitive function. Additionally, there was a significant positive relationship between patriarchal attitudes and cognitive impairment among the group with a good lifestyle.

Whether norms affect cognitive function has been studied with more focus on attitudes toward gender roles than on patriarchal norms. Several previous studies support our findings [40-44]. According to a previous study from the United States [40], men with strong conservative views regarding gender roles and a high degree of qualities aligned with traditional masculinity are significantly more likely to commit suicide than those who do not have such views or qualities. Another cross-sectional study investigated the relationship between patriarchal attitudes and men’s health by comparing women’s homicide rates to men’s mortality rates [41]. They found that nations with high-levels of patriarchal attitudes were significantly associated with higher mortality rates for men. The results suggest that oppression and exploitation harm not only the oppressed, but also the oppressors. We can also infer that highly gender-typed people are more likely to have mental disorders, such as depression and cognitive impairment. In our study, men with conservative beliefs regarding GRS had a much higher OR for cognitive impairment than women with conservative beliefs regarding GRS. Unlike conservative women, men with conservative attitudes toward GRS are more likely to have qualities aligned with traditional masculinity, such as competitiveness, emotional restriction, and aggression [45]. As a result, men with conservative beliefs regarding GRS may suffer negative effects from both GRS and a high degree of traditional masculinity. This can lead to a higher risk of cognitive impairment than women. An earlier case-control study investigated the effects of GRS on cognitive performance [42]. This study found that men with conservative attitudes toward gender stereotypes had significantly lower verbal fluency abilities than the control group, and even conservative women. From a young age, men and women are conditioned with gender stereotypes regarding what subjects they should excel in. For example, while boys are encouraged to excel at math, girls may be encouraged to excel at language skills. This gender stereotype could result in stress and affect mental health throughout men’s and women’s lives. Thus, the study suggested that their results may have been due to the threat posed by stereotypes on men’s verbal abilities. For another previous study, wholebrain functional magnetic resonance imaging was performed on 3 groups of women—positive-stereotype, negative-stereotype, and control groups [43]. Before undertaking the imagined self-rotation task, the positive-stereotype group was informed that women perform the task better than men do. The negative-stereotype group received the opposite message, and the control group was provided with neutral information. The results demonstrated that the positive-stereotype group made significantly fewer errors in performance than the control group, while the negative-stereotype group made significantly more errors than the control group. The results showed that gender stereotypes could substantially affect women’s cognitive performance. This study supports the findings of other studies that stereotypes could have a negative effect on mental health and cognitive function. A possible explanation based on neuroplasticity theory is that having such stereotypes decreases mental flexibility, resulting in impairments to general cognitive function. The importance of “openness” in learning has been well demonstrated in many studies on academic performance–personality relationships because of its correlation with positive learning approaches, learning motivation, and critical thinking [46-48]. Greater belief in stereotypical gender roles or patriarchal attitudes may indicate less openness, since new information can be overshadowed by stereotypical thinking for those with a strong belief in stereotypes [6]. For example, they may discount accomplishments that are inconsistent with stereotypical expectations [49]. As a result, people who tend to process only stereotypical information may become more vulnerable to cognitive impairment. Moreover, conservative attitudes toward GRS may prevent women from being educated or having an occupational role outside of performing housework, which may cumulatively affect their cognitive function [5].

Our study yielded several interesting findings. First, the group with weaker network constraints of spouse had a significant positive association between GRS and cognitive impairment. Stronger spouse constraints may be an obstacle that prevents a person from having an open mind to non-stereotypical gender roles. For example, a person with strong spouse constraints and their partner may be surrounded by a high-density social network. In such circumstances, even though one may not have a strong belief in GRS, one’s freedom to live in a way that goes against social stereotypes may be hindered. Secondly, GRS had a significant positive association with cognitive impairment in the older group, while patriarchal attitudes had a significant positive association with cognitive impairment in the younger group. This suggests that GRS and patriarchal attitudes mainly affect different age groups. This may be due to changes in spousal and familial relationships over time. Lastly, the model tested according to lifestyle suggests that bad lifestyles involving high levels of alcohol consumption and smoking may hinder potential cognitive impairment-preventive mechanisms.

This study has several strengths. First, we administered questionnaires by visiting participants’ homes individually. We obtained detailed and precise information on an individual level. Therefore, we could control for various potential confounders such as gender, age, household income, education, physical activity, depressive symptoms, social network size, smoking status, and drinking status. Second, our sample has unique characteristics. The study population consisted of people from a rural area, all of a single ethnic background, who formed a closed community within which most residents were born and raised, meaning that the homogeneity of the baseline population could be assumed. Therefore, our findings are unlikely to have been affected by various confounders such as residential area, ethnicity, or environmental factors.

Nonetheless, there are some limitations. First, since this was a cross-sectional study, we only determined the association of GRS and patriarchal attitudes with cognitive impairment, and we cannot suggest a causal relationship. Furthermore, among the group with cognitive impairment, around 76.2% did not have access to, and thus did not have the option to attend, elementary school. Based on this, we could consider the possibility of reverse causality—that is, participants’ low education level might affect their consistent conservative attitude regarding gender roles and patriarchal norms. Regardless, the significant association of GRS and patriarchal attitudes with cognitive impairment is an important finding in terms of the suggested effect gender equality could have on the prevention of cognitive impairment among the elderly. Second, we only assessed cognitive impairment using the MMSE-K test. However, the MMSE-K has been widely used as a diagnostic tool for assessing cognitive function in clinical practice and research. Furthermore, the MMSE-K has been proven to detect cognitive impairment well with high reliability and accuracy in actual public health settings. Because of the MMSE-K’s low cost and ease of use, it is recommended as the first step for measuring cognitive function and assessing cognitive impairment [50]. Third, we analyzed a study population from a single rural community. Even though we generated consistent results after replicating the analysis using data from another township, Township K and Township L were located on the same island. Moreover, the study participants were isolated on an island and were mainly engaged in farming and fishing. Their social communities may be more constrained than those of urban dwellers, and their lifestyles are also different from people living in urban areas. Thus, it might be difficult to generalize our results to other elderly populations. Finally, we excluded people diagnosed with severe cognitive impairment from the KSHAP survey, which may introduce selection bias. However, we measured cognitive function as a continuous variable using MMSE-K scores. Therefore, we could compare the degree of belief in stereotypical gender roles and patriarchal attitudes between people with lower and higher MMSE-K scores.

Our cross-sectional findings suggest that GRS and patriarchal attitudes may be independently associated with cognitive impairment in women later in life. We also found a significant association between patriarchal attitudes and cognitive impairment only in relatively younger people. Based on our results, we suggest that the value of gender equality and a more open mindset toward family members’ roles should be emphasized at the community level. Education should be provided to foster a more equal and less hierarchical culture for elderly communities. This study also suggests that the need to eliminate stereotypes is as important for the elderly population as it is for other age groups. Recognizing the implicit effects of stereotypes will lead people to have a more open-minded attitude toward gender roles and patriarchal norms and improve cognitive function in the elderly population.

In conclusion, our findings show a significant association of GRS and patriarchal attitudes with cognitive impairment; this is a novel finding in Asian countries with relatively conservative cultures. Studies on cognitive impairment require a multi-pronged approach on an individual, biological level as well as on a populational, psychosocial level. Therefore, this study is meaningful, as it confirmed a significant association reflecting East Asian culture and emotional customs. Further longitudinal studies are required to refine the mechanism and determine causality between stereotypical thinking and cognitive function.

## Figures and Tables

**Table 1.** General characteristics of the study population according to beliefs regarding gender role stereotypes and patriarchal attitudes

Characteristics	Total (n=580)	Gender role		p-value	Patriarchal attitude		p-value
		Open (n=256)	Conservative (n=324)		Weak (n=276)	Strong (n=304)	
Age (yr)	72.64±7.01	71.34±6.94	73.69±7.04	<0.001	71.54±7.22	73.57±6.65	<0.001
Gender, men	238 (41.0)	100 (39.1)	138 (42.6)	0.391	115 (41.7)	123 (40.5)	0.768
Household income (10^4^ KRW)							
<1,000	314 (54.1)	121 (47.3)	193 (59.6)	0.002	141 (51.1)	173 (56.9)	0.538
<2,000	124 (21.4)	66 (25.8)	58 (17.9)		66 (23.9)	58 (19.1)	
<3,000	53 (9.1)	25 (9.8)	28 (8.6)		26 (9.4)	27 (8.9)	
≥3,000	46 (7.9)	29 (11.3)	17 (5.3)		24 (8.7)	22 (7.2)	
Education							0.012
Less than elementary school	197 (34.0)	68 (26.6)	129 (39.8)	0.001	83 (30.1)	114 (37.5)	
Elementary school	224 (38.6)	102 (39.8)	122 (37.7)		101 (36.6)	123 (40.5)	
Middle school	79 (13.6)	42 (16.4)	37 (11.4)		41 (14.9)	38 (12.5)	
High school	55 (9.5)	26 (10.2)	29 (9.0)		33 (12.0)	22 (7.2)	
≥College	25 (4.3)	18 (7.0)	7 (2.2)		18 (6.5)	7 (2.3)	
Hypertension	284 (49.3)	117 (46.1)	167 (51.9)	0.167	140 (51.1)	144 (47.7)	0.413
Physical activity (times/wk)							
<1	280 (48.3)	110 (43.0)	170 (52.5)	0.080	133 (48.2)	147 (48.3)	0.248
1-4	66 (11.4)	32 (12.5)	34 (10.5)		35 (12.7)	31 (10.2)	
≥5	71 (12.2)	75 (29.3)	88 (27.2)		39 (14.1)	32 (10.5)	
Social network size							
≤3	353 (61.3)	151 (59.7)	202 (62.5)	0.485	168 (61.5)	185 (61.1)	0.905
>3	223 (38.7)	102 (40.3)	121 (37.5)		105 (38.5)	118 (38.9)	
Quality of relationship with spouse							
Weak	294 (50.7)	125 (48.8)	169 (52.2)	0.425	146 (52.9)	148 (48.7)	0.311
Strong	286 (49.3)	131 (51.2)	155 (47.8)		130 (47.1)	156 (51.3)	
Spouse constraints							
Weak	363 (62.6)	160 (62.5)	203 (62.7)	0.970	183 (66.3)	180 (59.2)	0.078
Strong	217 (37.4)	96 (27.5)	121 (38.4)		93 (33.7)	124 (40.8)	
Smoking status							
Former/non-smokers	522 (90.0)	230 (89.8)	292 (90.1)	0.911	243 (88.0)	279 (91.8)	0.134
Current smokers	58 (10.0)	26 (10.2)	32 (9.9)		33 (12.0)	25 (8.2)	
Drinking status							
Former/non-drinkers	469 (80.9)	213 (83.2)	256 (79.0)	0.203	218 (79.0)	251 (82.6)	0.274
Current drinkers	111 (19.1)	43 (16.8)	68 (21.0)		58 (21.0)	53 (17.4)	
Gender role score	6.77±1.72	8.33±1.13	5.54±0.97	<0.001	7.13±1.76	6.45±1.63	<0.001
Patriarchal mindset score	13.74±5.87	15.22±5.90	12.58±5.50	0.002	18.77±4.02	9.18±2.69	<0.001
CES-D	4 [1, 10]	3 [1, 9]	5 [2, 11]	<0.001	4 [1, 11]	4 [1, 10]	0.428
MMSE-K	25 [22, 27]	26 [23, 28]	24 [21, 27]	<0.001	26 [22, 28]	24 [21, 27]	0.024
MMSE-K, category							
Normal cognitive function^1^	437 (75.3)	216 (84.4)	221 (68.2)	<0.001	221 (80.1)	216 (71.1)	0.012
Cognitive decline^2^	143 (24.7)	40 (15.6)	103 (31.8)		55 (19.9)	88 (28.9)	

Values are presented as mean± standard deviation, median [interquartile range], or number (%). KRW, Korean won; CES-D, Center for Epidemiology Studies Depression scale; MMSE-K, Mini-Mental Status Examination, Korean version.

1 Normal cognitive function was defined as MMSE-K >21 points.

2 Cognitive decline was defined as MMSE-K ≤21 points.

## SUPPLEMENTARY MATERIALS

Supplementary Material 1. General characteristics of study population in men and women

Supplementary Material 2. Association between gender role stereotype and cognitive decline in study participants living in Township L

Supplementary Material 3. Association between gender role stereotype and continuous MMSE-K scores using linear regression analysis

Supplementary Material 4. Association between patriarchal mindset and continuous MMSE-K scores using linear regression analysis
