# Supplementary material for: Gender role stereotypes, patriarchal attitudes, and cognitive function in the elderly rural Korean population: a cross-sectional study
Source: Epidemiol Health. 2021 Apr 7;43:e2021023. doi: 10.4178/epih.e2021023 (PMC8289476; doi:10.4178/epih.e2021023)
Supplement: Supplementary Material 1. — General characteristics of study population in men and women [file epih-43-e2021023-suppl.DOCX]

| Supplementary Material 1. General characteristics of study population in men and women | | | | | | | | | | | |
| --- | --- | --- | --- | --- | --- | --- | --- | --- | --- | --- | --- |
| Variables | Total  (n=580) | | | Men (n=238) | | | Women (n=342) | | | p-value | |
| MMSE-K | 25 | [22, 27] | | 26 | [24, 28] | | 24 | [20, 27] | | <0.001 |  |
| Gender role score | 6.8 | ± | 1.7 | 6.6 | ± | 1.9 | 6.9 | ± | 1.6 | 0.096 |  |
| Patriarchal mindset score | 13.7 | ± | 5.9 | 14.0 | ± | 6.3 | 13.6 | ± | 5.5 | 0.442 |  |
| Age, yr | 72.6 | ± | 7.0 | 73.1 | ± | 6.4 | 72.3 | ± | 7.4 | 0.196 |  |
| Household income, KRW |  |  |  |  |  |  |  |  |  |  |  |
| < 10,000,000 | 314 | (54.1) | | 109 | (45.8) | | 205 | (59.9) | | <0.001 |  |
| < 20,000,000 | 124 | (21.4) | | 59 | (24.8) | | 65 | (19.0) | |  |  |
| < 30,000,000 | 53 | (9.1) | | 27 | (11.3) | | 26 | (7.6) | |  |  |
| ≥ 30,000,000 | 46 | (7.9) | | 29 | (12.2) | | 17 | (5.0) | |  |  |
| Education |  |  |  |  |  |  |  |  |  |  |  |
| Less than elementary school | 197 | (34.0) | | 21 | (8.8) | | 176 | (51.5) | | <0.001 |  |
| Elementary school | 224 | (38.6) | | 106 | (44.5) | | 118 | (34.5) | |  |  |
| Middle school | 79 | (13.6) | | 55 | (23.1) | | 24 | (7.0) | |  |  |
| High school | 55 | (9.5) | | 37 | (15.6) | | 18 | (5.3) | |  |  |
| College + | 25 | (4.3) | | 19 | (8.0) | | 6 | (1.8) | |  |  |
| Hypertension | 284 | (49.3) | | 97 | (41.1) | | 187 | (55.0) | | 0.001 |  |
| physical activity |  |  | |  |  | |  |  | |  |  |
| <1/week | 280 | (48.3) | | 104 | (43.7) | | 177 | (51.5) | | 0.013 |  |
| 1-4/week | 66 | (11.4) | | 30 | (12.6) | | 36 | (10.5) | |  |  |
| 5/week or more | 71 | (12.2) | | 41 | (17.2) | | 30 | (8.8) | |  |  |
| Social relationship |  |  |  |  |  |  |  |  |  |  |  |
| Social network size≤3 | 353 | (61.3) | | 136 | (57.6) | | 217 | (63.8) | | 0.134 |  |
| Social network size>3 | 223 | (38.7) | | 100 | (42.4) | | 123 | (36.2) | |  |  |
| Relationship with a spouse |  |  |  |  |  |  |  |  |  |  |  |
| Weak | 294 | (50.7) | | 82 | (34.5) | | 212 | (62.0) | | <0.001 |  |
| Strong | 286 | (49.3) | | 156 | (65.5) | | 130 | (38.0) | |  |  |
| Spouse constrain |  |  |  |  |  |  |  |  |  |  |  |
| Weak | 363 | (62.6) | | 131 | (55.0) | | 232 | (67.8) | | 0.002 |  |
| Strong | 217 | (37.4) | | 107 | (45.0) | | 110 | (32.2) | |  |  |
| Smoking |  |  |  |  |  |  |  |  |  |  |  |
| Former/Non-smokers | 522 | (90.0) | | 183 | (76.9) | | 339 | (99.1) | | <0.001 |  |
| Current smokers | 58 | (10.0) | | 55 | (23.1) | | 3 | (0.9) | |  |  |
| Drinking |  |  | |  |  |  |  |  |  |  |  |
| Former/Non-drinkers | 469 | (80.9) | | 143 | (60.1) | | 326 | (95.3) | | <0.001 |  |
| Current drinkers | 111 | (19.1) | | 95 | (39.9) | | 16 | (4.7) | |  |  |
| Values are shown as mean±SD, median[IQR], or number (%). | | | | | | | | | | | |

| Supplementary Material 2. Association between gender role stereotype and cognitive decline in study participants living in Township L | | | | | |
| --- | --- | --- | --- | --- | --- |
| Gender role | Case / no. of participants (%) | Odds ratio (95% CI) for dementia | | | |
|  |  | Unadjusted | | Multiple-adjusted* | |
| **Total (n=948)** |  |  | |  | |
| Conservative to gender roles | 157 / 685 (22.9) | 3.26 | (2.03-5.22) | 2.31 | (1.37-3.89) |
| Open to gender roles | 22 / 263 (8.4) | 1.00 | | 1.00 | |
| **Men (n=392)** |  |  | |  | |
| Conservative to gender roles | 47 / 246 (19.1) | 3.21 | (1.57-6.58) | 2.93 | (1.55-5.52) |
| Open to gender roles | 10 / 146 (6.9) | 1.00 | | 1.00 | |
| **Women (n=556)** |  |  | |  | |
| Conservative to gender roles | 110 / 439 (25.1) | 2.93 | (1.35-6.36) | 1.97 | (0.97-4.01) |
| Open to gender roles | 12 / 117 (10.3) | 1.00 | | 1.00 | |
| * adjusted for sex, age, household income, education, smoking and drinking status. | | | | | |

| Supplementary Material 3. Association between gender role stereotype and continuous MMSE-K scores using linear regression analysis | | | | | | | | | | | |
| --- | --- | --- | --- | --- | --- | --- | --- | --- | --- | --- | --- |
| **Gender role** | unadjusted | | |  | Model 1 | | |  | Model 2 | | |
|  | β (score) | SE | p value |  | β (score) | SE | p value |  | β (score) | SE | p value |
| **Total participants (n=580)** |  |  |  |  |  |  |  |  |  |  |  |
| Open | ref | | |  | ref | | |  | ref | | |
| Conservative | -1.830 | 0.395 | <0.001 |  | -1.223 | 0.349 | <0.001 |  | -0.934 | 0.428 | 0.030 |
| continuous, per 1 score decrease | -0.395 | 0.115 | <0.001 |  | -0.216 | 0.102 | 0.034 |  | -0.171 | 0.129 | 0.184 |
| **Men (n=238)** |  |  |  |  |  |  |  |  |  |  |  |
| Open | ref | | |  | ref | | |  | ref | | |
| Conservative | -0.963 | 0.448 | 0.033 |  | -0.668 | 0.434 | 0.125 |  | -0.885 | 0.498 | 0.077 |
| continuous, per 1 score decrease | -0.057 | 0.121 | 0.640 |  | -0.005 | 0.116 | 0.968 |  | -0.004 | 0.136 | 0.974 |
| **Women (n=342)** |  |  |  |  |  |  |  |  |  |  |  |
| Open | ref | | |  | ref | | |  | ref | | |
| Conservative | -2.562 | 0.566 | <0.001 |  | -1.587 | 0.498 | 0.002 |  | -0.904 | 0.650 | 0.166 |
| continuous, per 1 score decrease | -0.796 | 0.174 | <0.001 |  | -0.372 | 0.157 | 0.018 |  | -0.305 | 0.208 | 0.145 |
| Model 1: adjusted for sex and age | | | | | | | | | | | |
| Model 2: adjusted for sex, age, hypertension, household income, education, physical activity, social relationship, smoking and drinking status. | | | | | | | | | | | |

| Supplementary Material 4. Association between patriarchal mindset and continuous MMSE-K scores using linear regression analysis | | | | | | | | | | | |
| --- | --- | --- | --- | --- | --- | --- | --- | --- | --- | --- | --- |
| **Patriarchal mindset** | unadjusted | | |  | Model 1 | | |  | Model 2 | | |
|  | β (score) | SE | p value |  | β (score) | SE | p value |  | β (score) | SE | p value |
| **Total participants (n=580)** |  |  |  |  |  |  |  |  |  |  |  |
| Open | ref | | |  | ref | | |  | ref | | |
| Conservative | -0.899 | 0.398 | 0.024 |  | -0.231 | 0.349 | 0.509 |  | -0.115 | 0.418 | 0.784 |
| continuous, per 1 score decrease | -0.083 | 0.034 | 0.015 |  | -0.028 | 0.030 | 0.352 |  | -0.036 | 0.034 | 0.294 |
| **Men (n=238)** |  |  |  |  |  |  |  |  |  |  |  |
| Open | ref | | |  | ref | | |  | ref | | |
| Conservative | -0.801 | 0.444 | 0.073 |  | -0.442 | 0.432 | 0.307 |  | -0.462 | 0.488 | 0.345 |
| continuous, per 1 score decrease | -0.080 | 0.035 | 0.023 |  | -0.048 | 0.034 | 0.167 |  | -0.058 | 0.037 | 0.126 |
| **Women (n=342)** |  |  |  |  |  |  |  |  |  |  |  |
| Open | ref | | |  | ref | | |  | ref | | |
| Conservative | -0.920 | 0.580 | 0.113 |  | -0.161 | 0.501 | 0.749 |  | -0.012 | 0.620 | 0.985 |
| continuous, per 1 score decrease | -0.074 | 0.052 | 0.160 |  | -0.028 | 0.045 | 0.529 |  | -0.043 | 0.054 | 0.429 |
| Model 1: adjusted for sex and age | | | | | | | | | | | |
| Model 2: adjusted for sex, age, hypertension, household income, education, physical activity, social relationship, smoking and drinking status. | | | | | | | | | | | |
